# Evaluating molecular epidemiology of carbapenem non-susceptible *Klebsiella pneumoniae* isolates with MLST, MALDI-TOF MS, PFGE

**DOI:** 10.1186/s12941-023-00640-9

**Published:** 2023-10-27

**Authors:** Yunus Emre Ibik, Nebahat Ejder, Elif Sevim, Erva Rakici, Elif Seren Tanriverdi, Aysegül Copur Cicek

**Affiliations:** 1 Microbiology Laboratory, Ordu University Training and Research Hospital, Ordu, 52000 Turkey; 2 Department of Medical Microbiology, Faculty of Medicine, Recep Tayyip Erdogan University, Rize, Turkey; 3 Department of Medical Biology, Faculty of Medicine, Ahi Evran University, Kırşehir, Turkey; 4 Microbiology Laboratory, Malatya Training and Research Hospital, Malatya, Turkey; 5 Department of Medical Microbiology, School of Medicine, Istanbul Medipol University, Istanbul, Turkey

**Keywords:** *K. pneumoniae*, Carbapenem, PFGE, MLST, MALDI-TOF MS

## Abstract

**Background:**

This study aimed to evaluate antibiotic resistance genes and virulence genes and the clonal relationship of the carbapenem-nonsusceptible *Klebsiella pneumoniae* strains by molecular methods which are isolated from various clinical specimens from patients treated in tertiary care hospital in Turkey.

**Methods:**

Identification of 32 carbapenem non-susceptible *K. pneumoniae* were determined by VITEK-2 (BioMérieux, France) automated system. Thirteen colistin-resistant strains were tested with the broth microdilution method. Various antibiotic resistance genes and virulence genes frequently seen in carbapenem-resistant strains were screened by PCR. Immunochromatographic tests used in the rapid diagnosis of carbapenemases were compared with PCR results. In addition, PFGE, MLST and MALDI-TOF MS methods were used to determine the clonal relationship among these strains.

**Results:**

PCR demonstrated that 31 of the strains carried at least one of the carbapenemase genes. In one strain, the coexistence of *bla*_OXA−48+NDM_ was shown. The most common resistance genes were determined as *bla*_SHV_ (84.3%), *bla*_CTX−M−1_ (46.8%), *bla*_OXA−48_ (40.6%), *bla*_KPC_ (40.6%), *bla*_TEM_ (31.2%), *bla*_NDM_ (18.8%) respectively. Among the virulence genes; *magA* (68.7%) was the most common, followed by *kpn *(59.3%) and K2 (9.3%). Immunochromatographic tests were found to be 100% compatible with PCR results. All colistin-resistant isolates were also found to be resistant by colistin broth microdilution. In PFGE analysis, 25 different genotypes were determined and clustering isolates were collected in 5 different clusters and the clustering rate was 35.4%. In MLST analysis, ST101 type was determined as the most common ST type with a rate of 29%. ST101 is followed by ST16, ST307, ST14, ST147, ST309, ST377, ST395 and ST2096, respectively. The compatibility rate between MALDI-TOF MS and VITEK-2 was found 94.3%, in bacterial identification. In MALDI-TOF MS typing, the maximum similarity between the strains was less than 70% and clustering not shown.

**Conclusion:**

In addition to OXA-48, which is endemic in our country, it has been determined that KPC, which is more common in the world, is becoming increasingly common in our region. ST101 type was determined as the most common type between the strains. To the best of our knowledge, this is the first study that compares these three methods in our country. There may be differences between bacterial identifications made with VITEK-2 and MALDI-TOF MS. In this study, it was observed that MALDI-TOF MS analyses were not compatible with the typing of strains according to PFGE and MLST analysis results.

**Supplementary Information:**

The online version contains supplementary material available at 10.1186/s12941-023-00640-9.

## Introduction

*Klebsiella pneumoniae* is a Gram-negative opportunistic pathogen that causes healthcare-associated infections in hospitalized and immunocompromised individuals. *K. pneumoniae* causes urinary tract infection, pneumonia, sepsis and liver infections and is one of the leading causes of sepsis [1, 2]. It has recently acquired additional genetic features; The number of severe infections due to *K. pneumoniae* has increased and the efficacy of treatment decreased, due to the emergence of antibiotic-resistant or hypervirulent *K. pneumoniae* strains [3]. *K. pneumoniae* has a high resistance rate to many antibiotic classes such as β-lactams, fluoroquinolones and aminoglycosides [4].

Antimicrobial resistance is commonly associated with the spread of transmissible plasmids which occur through horizontal gene transfer. These plasmids can also carry virulence factors [5]. Capsule, lipopolysaccharide (LPS), fimbriae (type 1 and 3) and siderophores are virulence factors that contribute to *K. pneumoniae* pathogenicity [2]. The emergence of increased virulence in carbapenem-resistant *K. pneumoniae* (CRKP) strains is of concern. A new hypervirulent clinical variant of *K. pneumoniae* has emerged in recent years. It shows various clinical and bacterial phenotypic features. These features are seen in ambulatory patients, causing infection in unusual infection sites (e.g. liver, eye, cerebrospinal fluid), ability to metastatic spread and production of hypermucoviscous colonies in media [6].

In this study, carbapenem non-susceptible *K. pneumoniae* strains from various clinical specimens from patients treated in our hospital were isolated. In these strains, it was aimed to investigate the antibiotic resistance genes and virulence genes and the clonal relationship between the strains by molecular methods together with epidemiological data.

## Materials and methods

### Study isolates

In our study, 32 *K. pneumoniae* isolates, which were isolated from various clinical specimens at Recep Tayyip Erdoğan University Training and Research Hospital between December 2017 and December 2020, were included. For this study, permission was obtained from the Non-Interventional Clinical Research Ethics Committee of Recep Tayyip Erdogan University with the decision no: 2020/44 and the date; 30.04.2020, and the study was conducted in accordance with the Declaration of Helsinki (2008).

Identification and antibiotic susceptibility tests of the isolates were studied by VITEK-2 (BioMérieux, France) and determined moderately sensitive or resistant to at least one of the carbapenem (ertapenem, meropenem, imipenem) antibiotics. Strains stored at -80 °C in LB medium containing 20% glycerol were revived, and their carbapenem susceptibility was checked with 10 µg meropenem and 10 µg imipenem disc (Oxoid, Thermo, USA) according to European Committee on Antimicrobial Susceptibility Testing (EUCAST) breakpoints [7, 8].

### Detection of antibiotic resistance and virulence genes

DNA isolation for PCR to determine β-lactamase genes was performed by boiling method [9]. The most common β-lactamase genes in clinical samples were investigated by in-house PCR method. The list of primer sequences and PCR product sizes are shown in Table 1.

**Table 1:** Primers for Antibiotic Resistance

Primers	Primer Sequence (5´→ 3´)	Amplicon Size (bp)	Tm (°C)	References
*bla*_TEM_	F: AGTATTCAACATTTYCGTGT R: TAATCAGTGAGGCACCTATCTC	847	56	[10]
*bla*_SHV_	F: ATGCGTTATATTCGCCTGTG R: TTAGCGTTGCCAGTGCTC	843	55	[10]
*bla*_CTX−M−1_	F: GCGTGATACCACTTCACCTC R: TGAAGTAAGTGACCAGAATC	260	55	[10]
*bla*_CTX−M−2_	F: TGATACCACCACGCCGCTC R: TATTGCATCAGAAACCGTGGG	341	55	[10]
*bla*_GES_	F: ATGCGCTTCATTCACGCAC R: CTATTTGTCCGTGCTCAGGA	863	56	[10]
*bla*_PER_	F: ATGAATGTCATCACAAAATG R: TCAATCCGGACTCACT	927	50	[10]
*bla*_KPC_	F: ATGTCACTGTATCGCCGTCT R: TTTTCAGAGCCTTACTGCCC	893	55	[11]
*bla*_IMP_	F: CATGGTTTGGTGGTTCTTGT R: ATAATTTGGCGGACTTTGGC	488	56	[10]
*bla*_VIM_	F: ATTGGTCTATTTGACCGCGTC R: TGCTACTCAACGACTGAGCG	780	58	[10]
*bla*_NDM_	F: GAGATTGCCGAGCGACTTG R: CGAATGTCTGGCAGCACACTT	497	57	[10]
*bla*_OXA−48_	F: GCGTGGTTAAGGATGAACAC R: CATCAAGTTCAACCCAACCG	438	52	[11]

Some gene regions of virulence factors, which are common especially in hypervirulent strains, were investigated by in-house PCR method. The list of primer sequences, PCR product sizes are shown in Table 2. In addition, the String test showing hypermucoviscosity was performed on all strains [12].

**Table 2:** Primers for virulence

Primers	Primer Sequence (5´→ 3´)	Amplicon Size (bp)	Tm (°C)	References
*magA*	F: GGTGCTCTTTACATCATTGC R: GCAATGGCCATTTGCGTTAG	1282	58	[13]
*rmpA*	F: ACTGGGCTACCTCTGCTTCA R: CTTGCATGAGCCATCTTTCA	535	50	[13]
*kpn*	F: GTATGACTCGGGGAAGATTA R: CAGAAGCAGCCACCACACG	626	55	[13]
*allS*	F: CCGAAACATTACGCACCTTT R: ATCACGAAGAGCCAGGTCAC	508	58	[13]
*iutA*	F:GGCTGGACATCATGGGAACTGG R: CGTCGGGAACGGGTAGAATCG	300	66	[13]
*iucA*	F: AATCAATGGCTATTCCCGCTG R: CGCTTCACTTCTTTCACTGACAGG	239	59	[12]
K1	F:GTAGGTATTGCAAGCCATGC R: GCCCAGGTTAATGAATCCGT	1047	55	[14]
K2	F: GACCCGATATTCATACTTGACAGAG R:CCTGAAGTAAAATCGTAAATAGATGGC	641	58	[3]
K5	F: TGGTAGTGATGCTCGCGA R: CCTGAACCCACCCCAATC	280	58	[3]
K20	F: CGGTGCTACAGTGCATCATT R: GTTATACGATGCTCAGTCGC	741	58	[3]
K54	F: CATTAGCTCAGTGGTTGGCT R: GCTTGACAAACACCATAGCAG	881	58	[3]
K57	F: CTCAGGGCTAGAAGTGTCAT R: CACTAACCCAGAAAGTCGAG	1037	58	[3]

PCR screenings were accomplished in a final volume of 25 µL with 1 µL of DNA. The master mixture was composed of 1X buffer, 3 mM MgCl_2_, 200 µM dNTPs, 0.2 µM primers each and 2.5 U DNA Taq polymerase (Thermo Scientific, USA). PCR reactions were conducted according to the references. PCR products were run on a 1% agarose gel at 100 V for 60 min and visualized under UV light transilluminator (DNR Bio-Imaging System MiniLumi, Israel).

### Immunochromatographic test (ICT) and colistin broth microdilution

Twelve strains were examined in accordance with the manufacturer’s recommendations with the 12 RESIST-4 O.K.N.V. and IMP K-set (Coris BioConcept, Belgium) immunochromatographic test kits. OXA-48, KPC, NDM, VIM and IMP carbapenemases were examined with this test.

Thirteen isolates that were found to be resistant to colistin according to the VITEK-2 antibiogram results were studied with the Colistin MIC Plate Kit (Diagnostics, Slovakia) in accordance with the manufacturer’s recommendations. *Escherichia coli* ATCC 25,922 strain was used in the control well.

### Pulsed field gel electrophoresis (PFGE)

In our study, Durmaz et al. PFGE protocol was applied [15]. All strains were subjected to PFGE analysis with the CHEF-DR II machine (Bio-Rad, California, USA). *K. pneumoniae* ATCC 13,883 strain was used as the molecular marker. Gel images and DNA band profiles were analyzed using the GelCompar II software system (version 6.6; Applied Maths, Sint-Martens-Latem, Belgium) (optimization 1.0, tolerance 1.0, cut off %90). Dice correlation coefficient was used to calculate similarity for band analysis and UPGMA (Unweighthed Pairwise Grouping Mathematical Avenaging) was used for cluster analysis.

### Multilocus sequence typing (MLST)

Multilocus sequence typing of *K. pneumoniae* isolates that are not susceptible to carbapenem was determined by phylogenetic analyzes according to the protocols available on the Pasteur Institute website and databases (http://bigsdb.pasteur.fr/klebsiella/). Seven housekeeping genes, including *gapA, infB, mdh, pgi, phoE, rpoB* and *tonB* were amplified by PCR and DNA sequence analyzes were performed [1, 16]. According to the sequence results, allele numbers and sequence types were determined using the MLST website (https://bigsdb.pasteur.fr/klebsiella/klebsiella.html).

### Matrix-assisted laser desorption ionization time of flight mass spectrometry (MALDI-TOF MS)

Thirty two isolates were studied with MALDI-TOF MS (BioMérieux, France). Bacteria were identified by comparing the obtained mass spectra with the spectra in the V3.0 information database of the system. *E. coli* ATCC 8739 strain was used for system calibration and bacteria identification control. The result of the first test with VITEK MS (BioMérieux, France) with a confidence interval of ≥ 90% at the species level was used.

The spectra obtained for each isolate on VITEK MS were compared with each other by Saramis (V4.16) software analysis and the dendrogram was generated (Cutoff %70).

### Discrimination power of methods

The mathematical formula developed by Hunter and Gaston was used to determine the separability index of the strains with the MLST and PFGE methods and to determine the discrimination power of the method [17].

$$D=1-\frac{1}{N\left(N-1\right)}\sum_{j=1}^{s}{x_j\left(x_j-1\right)}$$

D = Discrimination index.

N = Number of strains tested in the study.

S = Total number of types identified.

nJ = Number of strains of type J.

The discrimination index ranges from 0 to 1. The fact that this value is close to 1 indicates that the discriminative power of the method is high.

## Results

Initially 35 isolates were included in the study. Two of the isolates (isolate no 12 and 22) were found to be *Klebsiella variicola* in identification with MALDI-TOF MS, and one (isolate no 8) was excluded due to carbapenem susceptibility. The study continued with 32 isolates.

### Antimicrobial susceptibility

Of the 32 isolates included in the study, 14 (43.7%) were urine, 11 (34.3%) were blood and catheter, 7 (21.8%) were respiratory tract samples. Of the 32 isolated strains, 16 (50%) came from intensive care units (ICU), 10 (31.2%) from inpatient wards and 6 (18.8%) from outpatient clinics.

Antibiograms of 32 isolates included in the study were studied with the VITEK-2 and the results are listed in Table 3.

**Table 3:** Antimicrobial susceptibility of isolates

Antibiotics	Susceptible (%)	Intermediate (%)	Resistant (%)
Amikasin (AK)	12 (37.6)	14 (43.7)	6 (18.7)
Gentamicin (GN)	25 (78.1)	0	7 (21.9)
Tobramycin (TOB)	5 (15.6)	0	27 (84.4)
Ciprofloxacin (CIP)	2 (6.3)	0	30 (93.7)
Levofloxacin (LEV)	2 (6.3)	0	30 (93.7)
Ceftazidime (CAZ)	0	0	32 (100.0)
Imipenem (IPM)	0	7 (21.8)	25 (78.2)
Meropenem (MEM)	0	0	32 (100.0)
Cefepime (FEP)	0	0	32 (100.0)
Piperacillin/tazobactam (TZP)	0	0	32 (100.0)
Trimethoprim/sulfametoxazole (SXT)	13 (40.6)	0	19 (59.4)
Colistin (COL)	19 (59.3)	0	13 (40.7)

### Detection of antibiotic resistance and virulence genes

The results of PCR resistance, virulence gene screening and specimen types are listed in Table 4.

**Table 4:** Resistance, virulence genes detected by PCR and specimen types

Sample No	Resistance Genes	Virulence Gene	Specimen
1	OXA-48, SHV, CTX-M-1	*magA*	Tracheal aspirate
2	OXA-48, SHV, CTX-M-1	*magA*	Tracheal aspirate
3	OXA-48, SHV, CTX-M-1	*magA*	Catheter
4	OXA-48, SHV, CTX-M-1	*magA*	Sputum
5	OXA-48, SHV, CTX-M-1	*magA*	Tracheal aspirate
6	OXA-48, SHV, CTX-M-1	*magA*	Blood
7	OXA-48, SHV, CTX-M-1	*magA, kpn*, K2	Blood
9	KPC, SHV	*kpn*	Blood
10	KPC, SHV	*kpn*	Blood
11	OXA-48, SHV, CTX-M-1	*magA*	Urine
13	OXA-48, SHV, CTX-M-1 TEM	*magA*, K2	Urine
14	OXA-48, SHV	*magA*	Urine
15	NDM, SHV, CTX-M-1, TEM	*magA,kpn*	Blood
16	KPC, SHV, TEM	*magA,kpn*	Tracheal aspirate
17	KPC, SHV, TEM	*magA,kpn*	Blood
18	KPC, SHV, TEM	*magA,kpn*	Tracheal aspirate
19	KPC, SHV	-	Urine
20	OXA-48, NDM, SHV	-	Blood
21	OXA-48, CTX-M-1	K2	Blood
23	KPC, SHV	*kpn*	Urine
24	KPC, TEM	*magA,kpn*	Blood
25	KPC, TEM, SHV	*magA,kpn*	Blood
26	KPC, TEM, SHV, CTX-M-1	*magA,kpn*	Urine
27	NDM, CTX-M-1, TEM	*kpn*	Urine
28	SHV, CTX-M-1	*magA,kpn*	Urine
29	NDM, SHV, CTX-M-1	*magA,kpn*	Urine
30	NDM, SHV	*magA,kpn*	Urine
31	KPC, TEM, SHV, CTX-M-1	*magA,kpn*	Sputum
32	NDM, SHV, CTX-M-1	*kpn*	Urine
33	KPC, SHV	*kpn*	Urine
34	OXA-48	*magA*	Urine
35	KPC, SHV	*kpn*	Urine

In all strains included in the study, *bla*_OXA−48_ was detected in 13 (40.6%) isolates, *bla*_KPC_ 13 (40.6%) and *bla*_NDM_ 6 (18.8%) isolates. No carbapenemase genes were detected in one strain. The one isolate coproducing OXA48 + NDM was isolated from blood culture of o woman in internal medicine ward. Among the other antibiotic resistance genes, *bla*_SHV_ was detected in 27 (84.3%), *bla*_CTX−M−1_ in 15 (46.8%) and *bla*_TEM_ in 10 (31.2%) isolates. Other resistance genes were not detected. Among the virulence genes, *magA* was detected the most in 22 isolates with a rate of 68.7%, while *kpn* was detected in 19 (59.3%) and K2 3 (9.3%) isolates. Other virulence factors were not detected. Isolates 19 and 26 exhibit positive string test.

### ICT and colistin broth microdilution

Twelve isolates were studied with RESIST-4 O.K.N.V. and IMP K-set (Coris BioConcept, Belgium) immunochromatographic assay kit. Carbapenemases that we evaluated as positive in this test were found to be 100% compatible with PCR results. The one strain that does not contain carbapenemase according to PCR results, also was not detected by ICT. Although there were MIC differences between VITEK-2 and colistin broth microdilution in colistin-resistant isolates, they were found to be 100% compatible in terms of sensitivity.

### PFGE

**Fig. 1:**
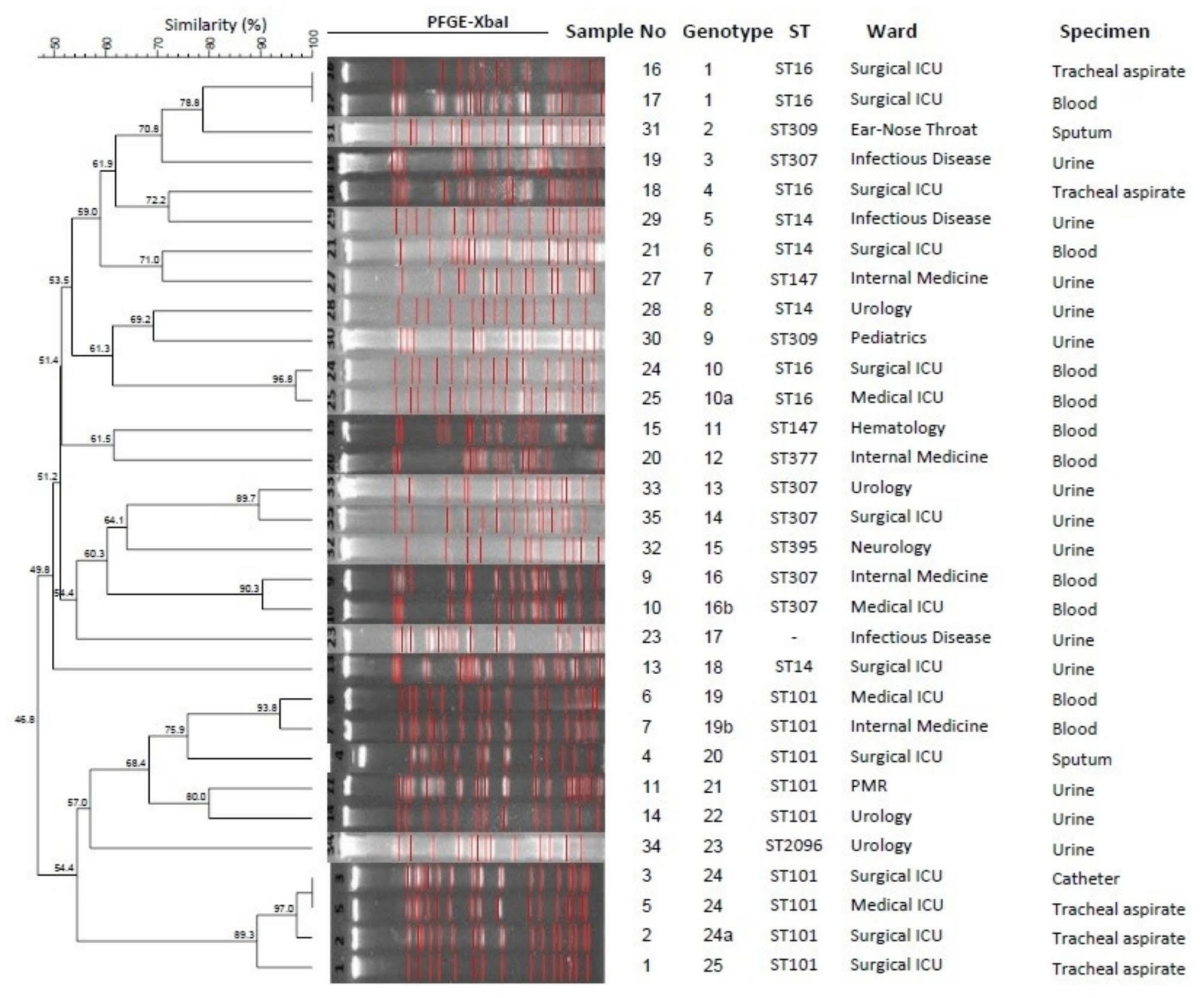
PFGE Dendrogram analysis. For each isolate typed by MLST, their respective sequence types (STs) and wards are represented. (*ST: Sequence type, ICU:Intensive Care Unit, PMR: Physical Medicine and Rehabilitation*)

One of the strains (Isolate no 26) could not be included in the study because band images could not be obtained due to the mucoid colony structure despite repeated studies. PFGE dendrogram analysis and epidemiological data of isolates were shown in Fig. 1.

Among 31 *K. pneumoniae* isolates, 25 different genotypes were detected, and clustered isolates were collected in 5 different clusters (tolerance 1.0, optimization 1.0, cutoff 90%). Eleven isolates are in a cluster, with a clustering rate of 35.4%. The largest cluster is the genotype 24 cluster with 3 isolates. Other clusters are genotype 1, 10, 16 and 19 clusters which contain two isolates each.

### MLST

As a result of MLST analysis, one strain (Isolate no 23) was found as *K. quasipneumoniae* and could not be evaluated. The ST types of the remaining 31 isolates were determined. ST101 type was determined as the most common ST type with a rate of 29%. It is followed by ST16 (19.4%), ST307 (16.1%), ST14 (12.9%), ST147 (6.5%), ST309 (6.5%), ST377 (3.2%), ST395 (3.2%) and ST2096 (3.2%), respectively.

### MALDI-TOF MS

**Fig. 2:**
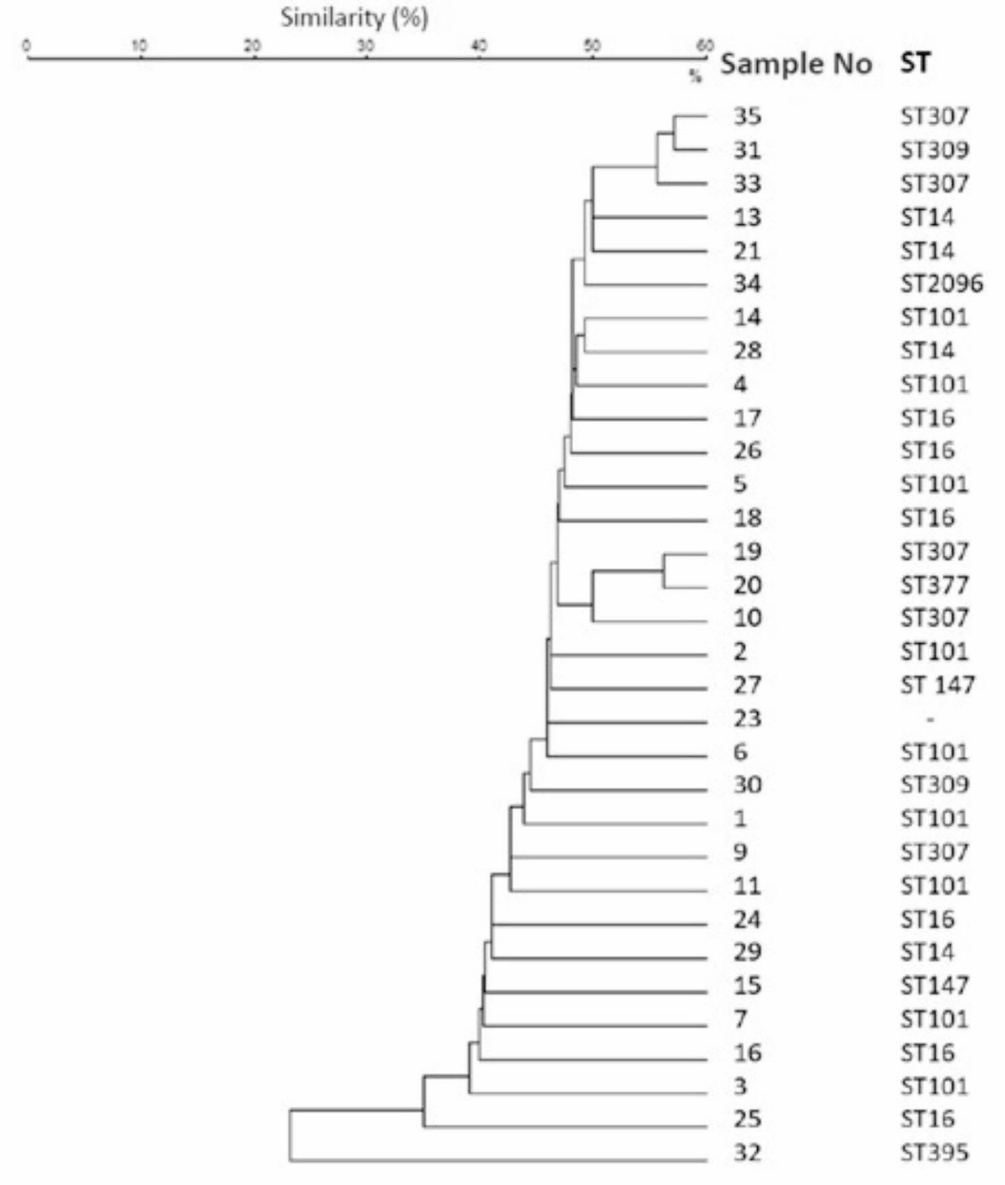
MALDI-TOF MS dendrogram analysis. Isolates’ respective sequence types (STs) are shown

MALDI-TOF MS identified two isolates (isolate no 12 and 22) as *K. variicola*, which were identified as *K. pneumoniae* by conventional methods and VITEK-2 automated system and were excluded from the study. As a result, compatibility rate between VITEK-2 identification and MALDI-TOF MS was found to be 94.3% in bacterial identification. In this case, 32 isolates were processed in the MALDI-TOF MS system. Dendrogram analysis based on mass spectra is given in Fig. 2.

As a result of the evaluation, the maximum similarity between the strains did not exceed 70% and clustering was not shown. There is a homogeneous distribution in the dendrogram. Carbapenem resistance did not cause any changes or differences in the peaks.

### Discrimination power of methods

The discriminatory power of the methods was determined as D = 0.9849 for PFGE and D = 0.8516 for MLST.

## Discussion

Antimicrobial resistance is increasing especially due to the inappropriate use of antibiotics and constitutes a global public health problem. It is widely known that hospital outbreaks, especially caused by multidrug-resistant *K. pneumoniae*, result in high morbidity and mortality due to limited treatment options [18, 19]. European Center for Disease Prevention and Control (ECDC) investigated the effect of antimicrobial resistance on the public health burden, it is estimated that the number of deaths attributed to infections due to CRKP increased 6-fold between 2007 and 2015 [20].

Recent studies show that resistance in *K. pneumoniae* isolates is increasing [21, 22]. Carbapenem resistance is generally seen together with aminoglycoside and quinolone resistance in Gram-negative bacteria [23]. In our study, while high resistance to quinolones was observed, aminoglycoside resistance was found to be relatively low. It is thought that this is due to the fact that the use of aminoglycosides is not preferred much in our hospital.

Various methods such as disk diffusion, E-test, automated systems, agar dilution and broth microdilution are used to detect colistin resistance. EUCAST recommends the broth microdilution method to measure colistin resistance. However, some studies have emphasized that the VITEK-2 system is also a reliable method for detecting colistin resistance [8, 24]. In our study, all strains found to be resistant with VITEK-2 were found to be resistant with broth microdilution. However, there are differences between MIC values.

Carbapenem resistance generally emerges with the transfer of *bla*_OXA−48_, *bla*_KPC_, *bla*_NDM_, *bla*_IMP_ and *bla*_VIM_ carbapenemase genes [25–28]. OXA-48 carbapenemase which was first detected sporadically in our country in 2001, then led to various epidemics and became endemic for our country [29, 30]. Studies show that OXA-48 is still the most common carbapenemase in our country [22, 31]. The first OXA-48 positive case of *K. pneumoniae*, which occurred outside of Turkey and was not clearly related to Turkey, was detected in a patient treated for lymphoma in Belgium in 2007 [32]. This gene is now also endemic in the Middle East and North African countries and causes nosocomial epidemics [33]. Studies conducted in our country showed that OXA-48 was found to be 68.8% and 82.45%, respectively, in CRKP isolates [26, 27]. Interestingly, this rate decreased to 40.6% in our study, and it was found at the same rate as KPC. *bla*_VIM_ and *bla*_IMP_, which are rare genes in our country, were not observed in our study [31, 34].

In our study, carbapenemase coexistence was detected as OXA-48 + NDM in only one strain. In the study of Cakar et al. in our country, the coexistence of OXA-48 and NDM was observed at a rate of 2.4% [35]. In another study conducted in our country, the association of KPC + OXA-48 was shown at a rate of 18% in CRKP isolates [36]. In the study of Genç et al., 1% coexistence of KPC + OXA-48 and 2% coexistence of KPC + NDM were shown [34].

In our country, there are few studies examining *K. pneumoniae* and virulence genes [37–39]. In our study, the presence of resistance gene and virulence genes were investigated in order to elucidate the underlying causes of *K. pneumoniae* isolates with multidrug resistance. *K. pneumoniae* has many virulence factors, especially capsular polysaccharide, hypermucoviscosity, fimbriae, toxins and iron uptake determinants [40]. In this study; *magA*, *rmpA*, *kpn*, *allS*, *iucA*, *iutA*, K1, K2, K5, K20, K54, K57 virulence genes were screened by PCR method. Selected virulence genes are kpn, the adhesin encoding gene; *rmpA*, mucoid regulatory phenotype A; *magA*, which is protectin or invasive mucoviscosity-related gene, *allS*; gene involved in allantoin metabolism; *iucA* and *iutA*, aerobactin receptor with siderophore gene; K1, K2, K5, K20, K54, K57 are genes associated with capsule proteins. As a result of PCR experiments, kpn was observed at a rate of 59.3%, *magA* at a rate of 68.7%, and K2 gene at a rate of 9.3%.

Carbapenem-resistant-hypervirulent *K. pneumoniae* strains result from horizontal transfer of resistance plasmids to hypervirulent *K. pneumoniae* strains. The emergence of these organisms can cause life-threatening infections in community [41]. *magA* causes increased capsule production and associated with hipervirulence [42]. String test shows hypermucoviscosity; the test is considered positive if the colony lengthens by 5 mm when the colony is drawn from the medium with the loop [43]. However, not all hypervirulent strains may have hypermucoviscousity [44]. While no virulence gene was detected in one of the two string test positive isolate, the other one showed *magA* and *kpn* positivity.

In a study conducted in our country, OXA-48, NDM and KPC production in 102 isolates of CRKP strains were examined with the ICT method with the RESIST-3 O.K.N K-SeT kit by Sağıroğlu et al. in 2018. When compared with PCR results, the sensitivity was found to be 100% [45]. In this study, RESIST-4 O.K.N.V. and IMP K-set immunochromatographic test results of 12 randomly selected isolates were found to be 100% compatible with PCR results. No positivity was detected in the isolate whose carbapenemase gene could not be detected by PCR.

MLST and PFGE are widely used in the epidemiological classification of bacteria. In addition, studies conducted in recent years have shown that MALDI-TOF MS has an important role in determining the clonal relationship in *K. pneumoniae* strains [46, 47].

Although PFGE, which creates genome-wide DNA fingerprints with restriction enzymes, is the gold standard in demonstrating the epidemiological relationship, it is labor intensive and may cause difficulties in comparisons between laboratories, especially due to user errors in identifying bands in PFGE gel image analysis [48]. While the power of the PFGE method for clonal typing and determining the source of epidemics is high in short-term outbreaks, it has been shown that this feature decreases in the long-term [49, 50]. In this study, 31 *K. pneumoniae* strains isolated between 2018 and 2020 were typed by PFGE and 25 types were determined. A significant part of the *K. pneumoniae* strains found in our hospital are clonally different and it suggests that the epidemics have many different origins. Of these patterns, cluster 24 formed the largest group with three samples. It was determined that isolates in the same genotype, the classical epidemiological data and the ST types were similar. Although the sample types of the two isolates included in genotype 1 were different, it was observed that they were obtained from patients hospitalized in the surgical ICU. The isolates in genotypes 10, 16 and 19 were isolated from blood culture. It was determined that three isolates in genotype 24 were obtained from catheter and tracheal aspirate samples isolated from intensive care units. These results show that although the aggregated isolates in our hospital did not cause an epidemic, they were mainly obtained from patients hospitalized in intensive care units, and it is important to follow-up this patient group in terms of infection control.

In long-term outbreaks, the PFGE patterns of isolates can change significantly over time and can be easily misinterpreted as a result. The combination of PFGE and MLST can confirm whether clonal spread has occurred and provide a better understanding of the evolution of epidemic isolates [51]. In a study by Sakai et al., molecular analysis was performed with PFGE to evaluate horizontal transmission during an outbreak of carbapenem-resistant *Enterobactericeae* in an intensive care unit, and MLST was used for analysis in addition to PFGE for some of these strains and the results were found to be compatible with each other [52]. In a study conducted in Italy, PFGE was shown to have a better discriminatory power when the distinguishing features of PFGE and MLST were compared with the number of unique STs and the number of clusters identified [48]. According to the PFGE analysis in our study, the STs of the isolates in the same cluster were found to be the same. This study confirmed that PFGE, which is the gold standard method, has higher discriminatory power as isolates with the same STs are located in different clusters. However, isolates with same sequence type showed close relation in PFGE analysis as you can see in Fig. 1.

The MLST method has also been increasingly used in recent years. In a study conducted in our country by Hazirolan et al. with 93 carbapenem and colistin-resistant *K. pneumoniae* strains, ST101 was the most detected clone in MLST analysis [53]. In another study, ST14 and ST2096 were seen as the dominant types, while OXA-48 positivity was detected in almost all of the *K. pneumoniae* strains [54]. In our study, ST101 was detected the most with a rate of 29%. The ST101 clone carries the plasmid IncL, which contains the *bla*_OXA−48_ gene, is responsible for the spread of carbapenemases [55]. All ST101 clones detected in our study carry the *bla*_OXA−48_ gene in parallel with this study.

The KPC-producing clone worldwide is *K. pneumoniae* ST258 [56]. ST11 is a single locus variant of ST258, and especially subtypes of KPC-2 and KPC-3 have been implicated in the global spread of KPC over the last 20 years and belong to the same clonal complex [57]. In our study, it was determined that the strains containing KPC mostly belonged to the ST16 and ST307 clones. In various studies, while the ST16 clone is mostly associated with OXA-48 and NDM, it is rarely associated with KPC [19, 58]. In addition, the rate of KPC in *K. pneumoniae* in our country was generally found at low rates or not shown [27, 34, 45, 53]. To the best of our knowledge, it is the highest rate of KPC (40.6%) detected in our country.

Traditional typing methods, MLST and PFGE, are very labor-intensive and relatively expensive techniques. However, MALDI-TOF MS provides fast results and a low-cost per sample analysis. MALDI-TOF MS typing is based on the expression of more conserved intrinsic proteins, which limits the evolutionary classification of strains [59].

It has been shown that MALDI-TOF MS can be used in the molecular typing of CRKP, including high efficiency and ease of use in a short time [60]. In a study conducted in China, MALDI-TOF MS and MLST methods were used together with 71 CRKP izolates. Almost all of the ST11 isolates had KPC-2. Nearly all ST11 isolates clustered in MS types which covers half of all MALDI-TOF MS types [1]. In a study by Fang et al. with *K. pneumoniae* producing KPC-2, good compatibility was demonstrated between PFGE and MALDI-TOF MS typing [60]. However, in this study, homogeneous distribution was observed in the dendrogram created with MALDI-TOF MS, no grouping was found and the similarity rate did not exceed 70%. Carbapenem resistance did not change the peaks, and this is thought to be due to the involvement of different genes in the formation of resistance such as *bla*_KPC_, *bla*_NDM_, *bla*_OXA−48_. Isolates 16 and 17 which have same pattern in PFGE analysis did not show close relation on MALDI-TOF MS dendrogram. Also isolates with the same ST types did not clustered on MALDI-TOF MS dendrogram unlike on PFGE dendrogram.

In this study, a high compatibility was detected between PFGE and MLST typing results, while the compatibility between MALDI-TOF MS and PFGE typing was low. Similarly, in other studies, MALDI-TOF-based typing was found to be less compatible with other reference methods (WGS, PFGE, MLST) used in the typing of *K.pneumoniae* [46, 61, 62].

There are very few studies comparing these three methods in the molecular typing of *K. pneumoniae* [46, 63]. To the best of our knowledge, this study is the first study conducted on this subject in our country.

*K. quasipneumoniae* and *K. variicola*, which were previously subtypes of *K. pneumoniae*, are now considered separate species. However, it is difficult to distinguish between these species using conventional diagnostic methods due to their close phenotypic and biochemical characteristics. In addition, the mass spectra generated by the analysis of these bacteria by MALDI-TOF MS are very similar to the spectrum of *K. pneumoniae* and that leads to misidendifications [64]. As a matter of fact, in this study, the two isolates detected as *K. pneumoniae* with VITEK-2 were identified as *K. variicola* with MALDI-TOF MS. In addition, another strain detected as *K. pneumoniae* with both VITEK-2 and MALDI-TOF MS was detected as *K. quasipneumonia*e in the analysis performed with MLST.

Limitations: only disc diffusion has been done for carbapenem resistance control in revived strains, colistin broth microdilution has been done only for **colistin**-resistant strains, not being able to perform ICT for all strains and not sending the detected genes to the sequence due to a lack of budget.

## Conclusion

In addition to OXA-48, which is endemic in our country, it has been determined that KPC, which is more common in the world, is becoming increasingly common in our region. ST101 type was determined as the most common type between the strains. MALDI-TOF MS is not a promising method for typing. There may be differences between bacterial identifications made with VITEK-2 and MALDI-TOF MS. In this study, it was observed that MALDI-TOF MS analyzes were not compatible with the typing of strains according to PFGE and MLST analysis results. Monitoring of carbapenem-resistant bacteria by each institution in terms of infection control will contribute to rational antibiotic use policies and infection control measures to combat antibiotic resistance, which has become a global public health problem all over the world.

### Electronic supplementary material

Below is the link to the electronic supplementary material.

Supplementary Material 1

Supplementary Material 2

Supplementary Material 3

Supplementary Material 4

## Data Availability

The data that support the findings of this study are available from the corresponding author upon reasonable request.

## List of abbreviations

AK: Amikasin
CAZ: Ceftazidime
CIP: Ciprofloxacin
COL: Colistin
CRKP: Carbapenem resistant *K. pneumoniae*
DNA: Deoxyribonucleic acid
EUCAST: European committee on antimicrobial susceptibility testing
FEP: Cefepime
GN: Gentamicin
Inc: Incompatibility
IPM: Imipenem
LEV: Levofloxacin
LPS: Lipopolysaccharide
MALDI-TOF MS: Matrix-assisted laser desorption ionization–time of flight mass spectrometry
MEM: Meropenem
MIC: Minimum inhibitory concentration
PCR: Polymerase chain reaction
PFGE: Pulsed-field gel electrophoresis
SXT: Trimetoprim/sulfametoxazol
ST: Sequence type
TIG: Tigecycline
TOB: Tobramycin
TZP: Piperacillin/tazobactam
