# Supplementary figures and images for: Evaluating molecular epidemiology of carbapenem non-susceptible Klebsiella pneumoniae isolates with MLST, MALDI-TOF MS, PFGE
Source: Ann Clin Microbiol Antimicrob. 2023 Oct 27;22:93. doi: 10.1186/s12941-023-00640-9 (PMC10612262; doi:10.1186/s12941-023-00640-9)

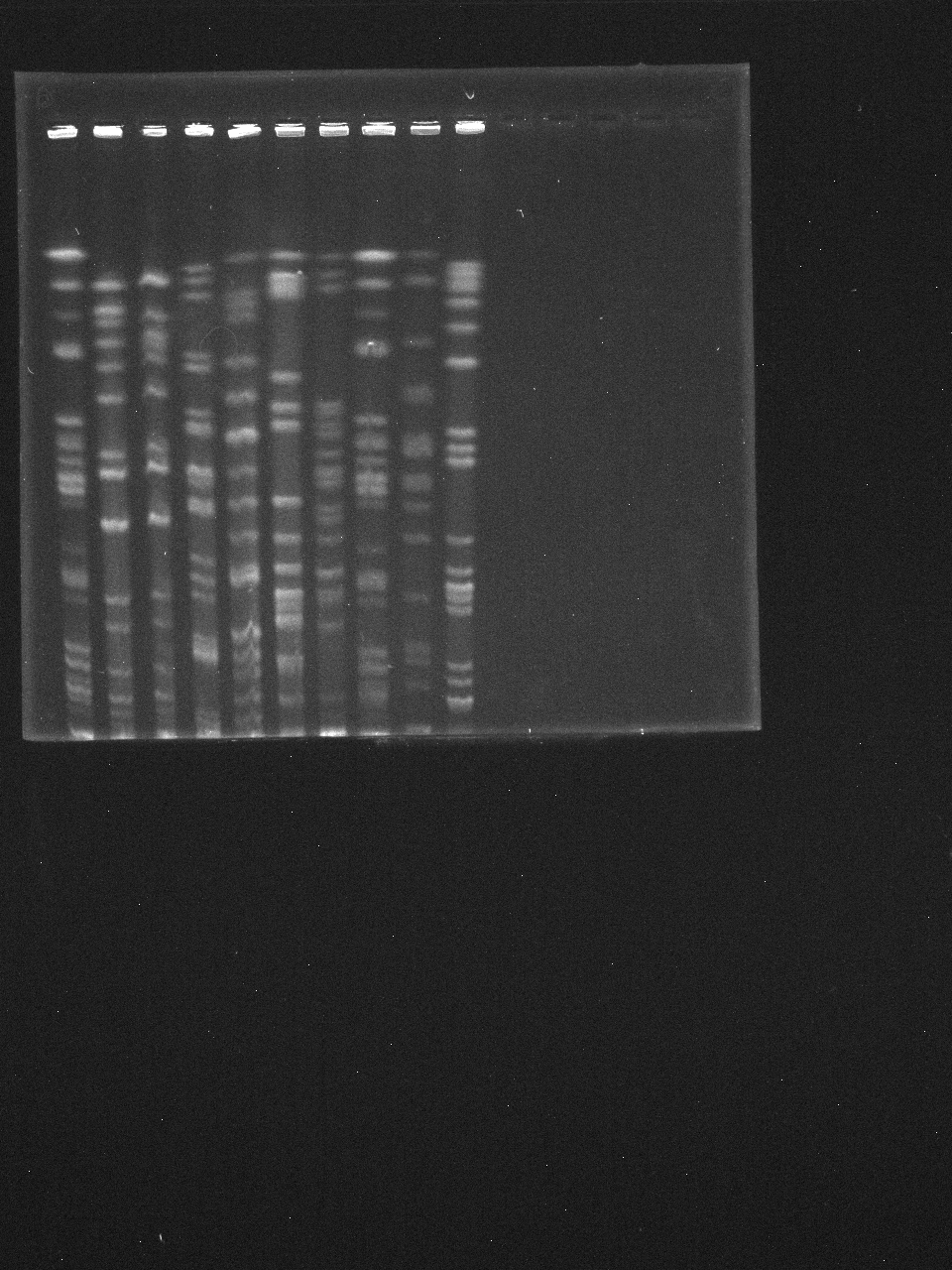

Supplement: Supplementary file 1 — Supplementary Material 1 [file 12941_2023_640_MOESM1_ESM.png]

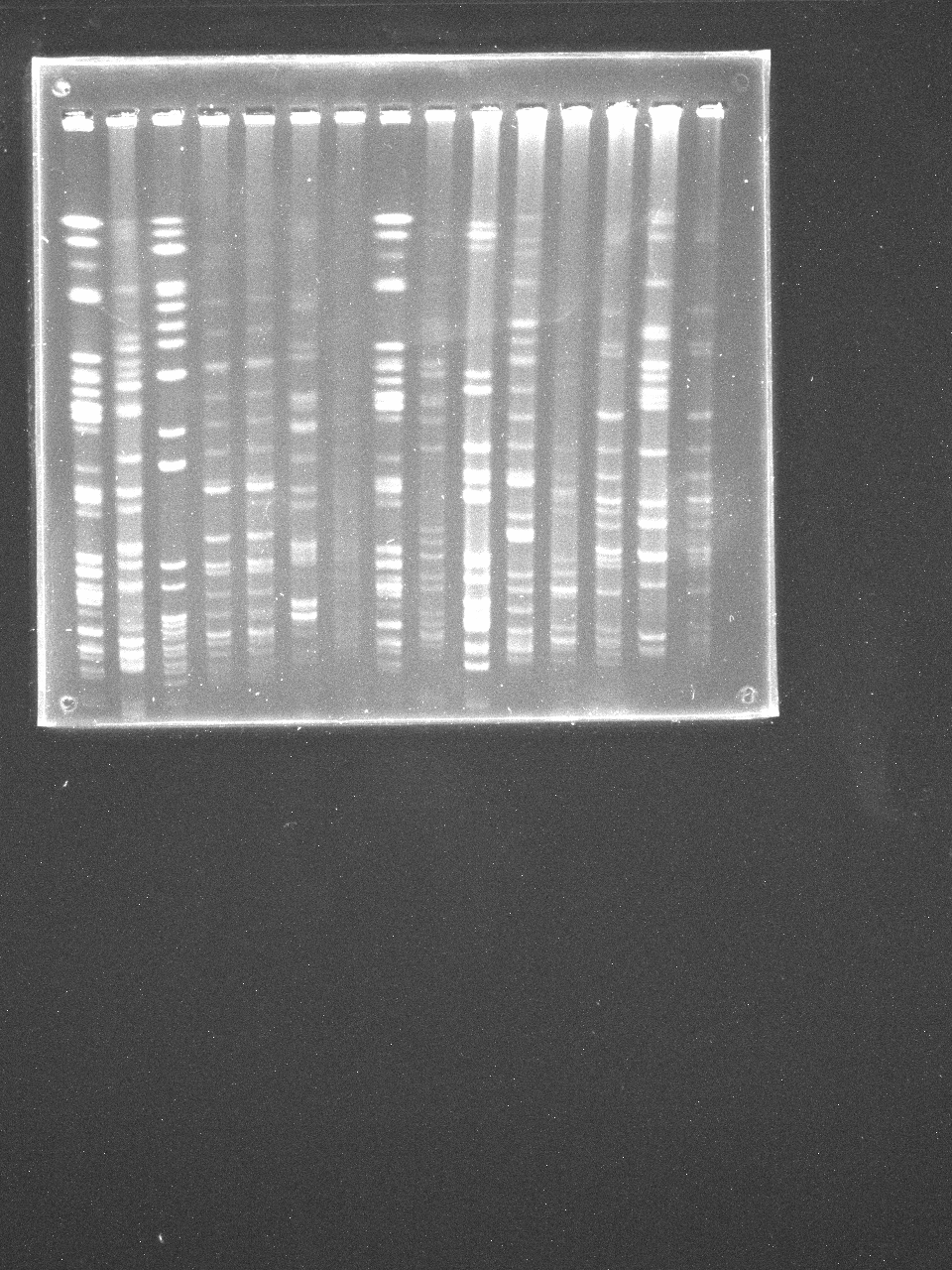

Supplement: Supplementary file 2 — Supplementary Material 2 [file 12941_2023_640_MOESM2_ESM.png]

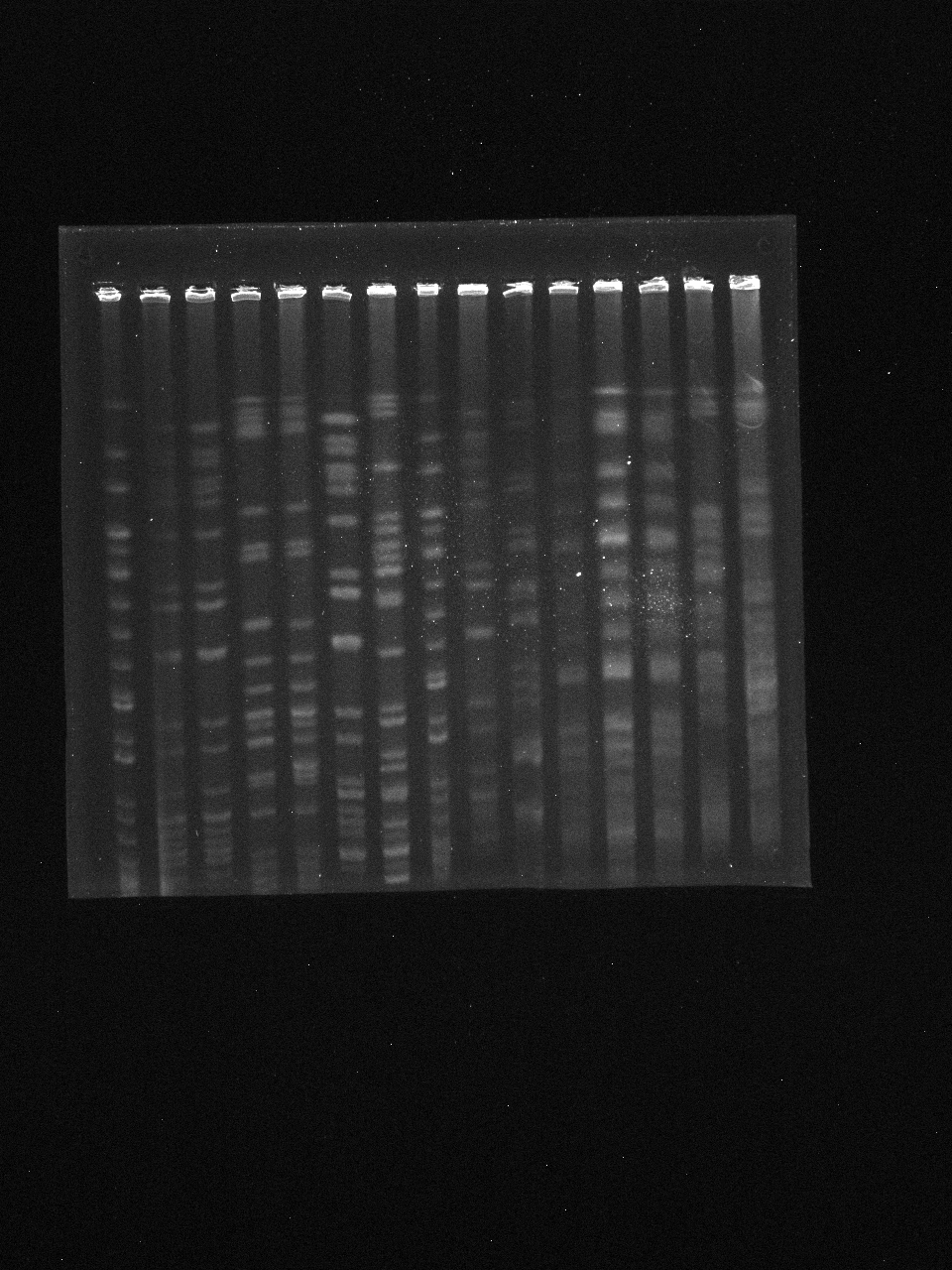

Supplement: Supplementary file 3 — Supplementary Material 3 [file 12941_2023_640_MOESM3_ESM.png]

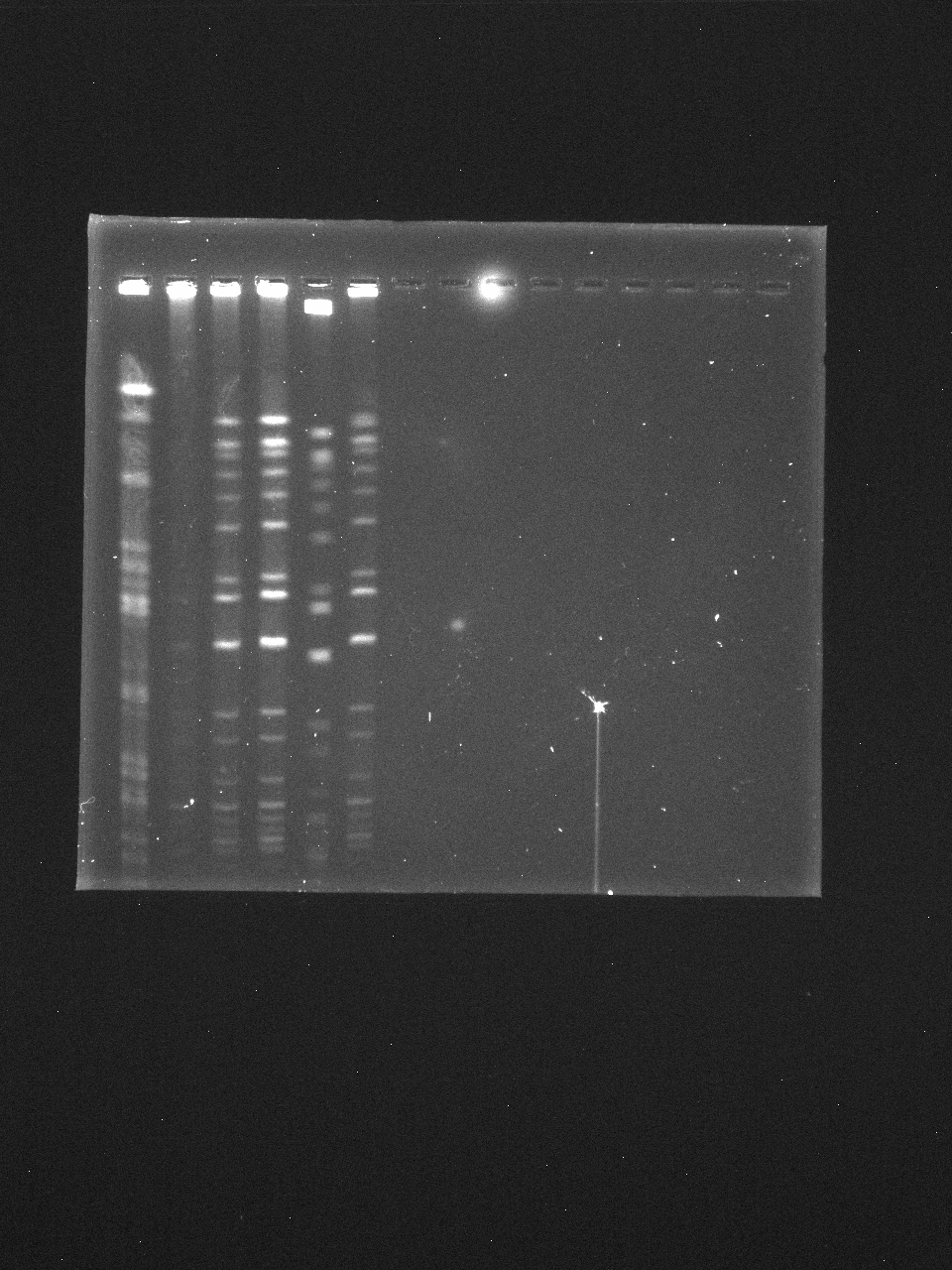

Supplement: Supplementary file 4 — Supplementary Material 4 [file 12941_2023_640_MOESM4_ESM.png]
